# Neurologists’ lived experiences of communicating the diagnosis of a motor neurodegenerative condition: an interpretative phenomenological analysis

**DOI:** 10.1186/s12883-023-03233-3

**Published:** 2023-05-03

**Authors:** Eleftherios Anestis, Fiona J. R. Eccles, Ian Fletcher, Jane Simpson

**Affiliations:** Division of Health Research, Faculty of Health and Medicine, Lancaster University, Lancaster, UK

**Keywords:** Breaking bad news, Neurodegenerative conditions, Motor neurone disease, Multiple sclerosis, Parkinson’s disease, Huntington’s disease, Doctor-patient communication, Interpretative phenomenological analysis

## Abstract

**Background:**

Receiving the diagnosis of a motor neurodegenerative condition (MNDC) can be a life-changing experience. Although several studies of individuals’ experiences have indicated dissatisfaction with aspects of how an MNDC diagnosis was communicated, few studies have addressed doctors’ experiences of breaking bad news for these conditions, especially from a qualitative perspective. This study explored UK neurologists’ lived experience of delivering an MNDC diagnosis.

**Methods:**

Interpretative phenomenological analysis was used as the overarching method. Eight consultant neurologists working with patients with MNDCs took part in individual, semi-structured interviews.

**Results:**

Two themes were constructed from the data: ‘Meeting patients’ emotional and information needs at diagnosis: a balancing act between disease, patient and organization-related factors’, and ‘Empathy makes the job harder: the emotional impact and uncovered vulnerabilities associated with breaking bad news’. Breaking the news of an MNDC diagnosis was challenging for participants, both in terms of achieving a patient-centred approach and in terms of dealing with their own emotions during the process.

**Conclusions:**

Based on the study’s findings an attempt to explain sub-optimal diagnostic experiences documented in patient studies was made and how organizational changes can support neurologists with this demanding clinical task was discussed.

**Supplementary Information:**

The online version contains supplementary material available at 10.1186/s12883-023-03233-3.

## Background

Breaking bad news has been recognised as a critical aspect of healthcare communication and one of the most difficult tasks doctors face [1]. Bad news has been commonly defined as “any news that drastically and negatively alters the patient’s view of his or her future” [2] such as the delivery of a medical diagnosis. Because how such news is delivered can have both a short- and long-term impact on patient outcomes [3–6] the topic has attracted considerable attention from healthcare and medical education researchers. Research on breaking bad news has mainly focused on patient perspectives and preferences and the development and evaluation of doctors’ training; however, doctors’ experiences and perspectives on breaking bad news have been less commonly investigated [7].

It is acknowledged that breaking bad news is a complex communication task that requires multiple competencies beyond just disclosing the name of a diagnosis. These can include delivering potentially distressing information in a sensitive way, assessing patients’ information needs and capability to absorb bad news, identifying and managing the emotional impact of bad news on all parties involved in the consultation, discussing prognosis and facilitating shared decision-making [8]. To meet patients’ preferences, doctors face the challenge of breaking bad news truthfully but also empathically, without taking away hope [9]. Despite the challenging nature of breaking bad news, a recent international survey of more than 10,000 healthcare professionals, including doctors, showed that only about a third had received formal training on breaking bad news [10]. It is, therefore, not surprising that doctors often feel they lack the necessary skills [11] and feel underprepared for the responsibility of breaking bad news [12, 13]. Moreover, studies have shown that breaking bad news can be a stressful task with stress reactions lasting beyond the actual consultation and potentially contributing to symptoms of burnout [14]. Doctors sometimes also fear eliciting strong emotional reactions from their patients or being blamed when breaking bad news [15] and can experience intense emotional reactions themselves such as guilt, failure and frustration [16].

Although most research on breaking bad news has been conducted within oncology, the importance of breaking bad news in other specialties, such as neurology, is increasingly recognised [17]. Motor neurodegenerative conditions (MNDCs), such as Parkinson’s disease (PD), multiple sclerosis (MS), Huntington’s disease (HD) and motor neurone disease (MND), are incurable and progressive, impacting patients’ movement, cognition and psychological functioning [18]. HD and MND are also more directly life-threatening, with MND patients’ life expectancy, for example, estimated at three years on average after symptom onset [19]. A review on patients’ perspectives of diagnosis delivery showed that receiving an MNDC diagnosis can be the end-product of a long and distressing process, with the diagnostic consultation an easily-recalled, critical and often shocking moment for patients [20]. This review also revealed mixed views on patients’ satisfaction with doctors’ approach to breaking bad news, with negative experiences highlighting inadequate information and time provision and lack of emotional support and sensitivity at diagnosis.

MNDCs are most commonly diagnosed by neurologists whose perspectives on breaking bad news have been sparsely addressed by research. The review also found very few studies of neurologists’ perceptions of breaking bad news; most were quantitative and focussed more on the parameters of their practice such as the terminology used and the timing of the diagnosis and less on their actual experience of breaking bad news. Further, a survey study on UK neurologists’ perspectives showed that most participants considered breaking bad news to be a difficult and stressful task, with being honest without taking away hope and spending the right amount of time the main challenges [21]. Although the survey was useful in that it identified several areas of improvement, it did not adequately capture neurologists’ in-depth experience of breaking bad news for MNDCs.

Capturing these experiences is an important part of narrative medicine, where doctors are considered to bring their own ‘stories’ to a consultation, based on their personality and their life and clinical experiences [22]. Addressing doctors’ subjectivity, understanding them as a person and not just as a skilled professional has been suggested as one of the key dimensions of patient-centredness [23]. Physicians deal with patients’ needs and expressed emotions using their own emotions, such as a need to ‘rescue’ the patient or feelings of failure, frustration and powerlessness when an illness is progressing or is untreatable [24]. Doctors’ emotions, their ‘inner life’, can thus have a crucial role in the doctor-patient interaction and the overall quality of care and when unexamined, emotions can affect doctors’ well-being and clinical judgment [24, 25]. For this reason, qualitative approaches are best suited to exploring the essentially subjective experience [26].

Consequently, the primary aim of this study was to explore UK neurologists’ lived experience of delivering an MNDC diagnosis and being the bearer of bad news, with the specific research question being: How do neurologists make meaning out of their experiences of breaking bad news and how is their practice shaped by this meaning-making process?

## Methods

### Study design and ethical approval

Interpretative phenomenological analysis (IPA) was used to inform the study design. IPA was chosen as it has been widely used in health research and its phenomenological, hermeneutical and idiographic underpinnings are well-suited for the study of lived experience [27]. The IPA approach is also aligned with critical realism, the researchers’ ontological and epistemological positioning which supports an ontological realism (there is a real world irrespective of human perception) and an epistemological relativism (an observable world is constructed from human perspectives and experiences and researchers have an active role in interpretating and producing knowledge) [28].

The semi-structured interview schedule was developed based on previous research on doctors’ perspectives on breaking bad news [16, 29], models of breaking bad news [9, 30], and a recent review of patients’ with MNDCs perspectives on diagnosis delivery [20]. Two neurologists gave initial feedback on the relevance and appropriateness of the questions and adjustments were made. The study received ethical approval by Lancaster University’s Faculty of Health and Medicine Ethics Committee and the Health Research Authority, a unified system for the governance of health research in the UK.

### Sampling and participants

Due to IPA’s idiographic nature and emphasis on detailing individual experiences and meaning-making processes, it is generally recommended that IPA studies have a homogenous and small sample size [31]. For this study, consultant neurologists who practised in the UK and delivered at least one of the diagnoses we focused on (PD, MS, MND or HD) were eligible for participation. To ensure the sample’s homogeneity, we chose to exclude neurologists in training as their experiences of breaking bad news were expected to be both quantitatively and qualitatively different. Participants were approached through collaborations with National Health Service neurology departments and centres for MNDCs and also through snowball sampling and advertisement of the study on social media.

Consent forms were signed and electronically submitted before interview. Eight neurologists took part in the study, a sample size within the recommended IPA sample sizes [32]. Most participants were male (*n* = 6), ages ranged from 38 to 54 years (M = 41) and years of experience as a consultant neurologist ranged from 2 to 20 (M = 10). We chose not to report the demographics of individual participants which could potentially include identifiable information.

### Data collection

All participants were interviewed by the first author, one interview was conducted in person and the rest over the phone or through video calls. Interviews lasted from 27 to 79 min (M = 52 min). Interviews were audio-recorded and transcribed verbatim by the first author. The interview schedule can be found as Supplementary Material 1.

### Data analysis

Data were analysed following the guidance and steps outlined by Murray and Wilde [32]. Familiarisation with the data was achieved by listening to and transcribing the interviews and reading the transcripts. Following an idiographic approach, each transcript was coded line-by-line using descriptive, interpretative and linguistic comments (coding examples and an audit trail of the analysis can be found as Supplementary Material 2). After coding was completed, codes were reviewed and patterns of meaning and themes related to the research aims were identified, summarised into text and given a title. This process was followed for every transcript before moving to the next. Finally, a cross-transcript analysis was completed by identifying points of convergence and divergence which allowed for a more complete and in-depth understanding of the research phenomenon. Quotes from individual interviews were then incorporated within these themes to evidence the interpretation and analysis.

### Quality, rigor and reflexivity

In order to ensure quality and rigor, both generic qualitative research and IPA-specific quality indicators were used during the entire research process. Rigor [33–35] was ensured through intense familiarisation with the transcripts, line-by-line coding and an in-depth idiographic analysis performed by the first author and discussions with the other authors. These discussions focussed on reviewing whether the coding and interpretations were rooted in the participants’ words and the overall context and content of their accounts. This is an appropriate approach and congruent with IPA [32]. When writing up the themes, special consideration was given to transparency [33, 34], helping the reader understand how interpretations were derived from the data (e.g., through the use of quotes) and producing themes with a coherent and compelling narrative addressing both convergences and divergences [36]. Moreover, reflexivity was an integral part of both data collection and analysis [37]. The researchers are not medical professionals but health researchers with expertise in the psychology of neurodegenerative conditions and substantial experience in conducting research with patients with MNDCs, including a scoping review on patients’ with MNDCs perspectives of diagnosis delivery which showed that patient experiences were often negative [20]. Through reflection, the lead author realised that this understanding could potentially narrow down the topics discussed during interviews which could appear as critical interrogations of neurologists’ practice of breaking bad news. Instead, acknowledging our outsider researcher positionality [38] in the medical field, a genuinely ‘curious’ attitude was maintained during the interviews which aimed at exploring doctors’ lived experiences and understand how their practice was shaped by their experiences and meaning-making processes. Also, considering the process of the ‘double hermeneutic’ in IPA (where the researcher tries to make sense of participants who are also trying to make sense of their experiences), coding, interpretations and theme development were completed through a process of ensuring constant and ‘close proximity to the data’ [35] and acknowledging the role of the researchers’ preconceptions in the analysis. This was intended to minimise the researchers’ preconceptions and produce interpretative accounts grounded in participants’ understandings and meanings of their experiences [31].

## Results

Two themes were developed which focussed on different aspects of participants’ experience of breaking bad news: ‘Meeting patients’ emotional and information needs at diagnosis: a balancing act between disease, patient and organization-related factors’ and ‘Empathy makes the job harder: the emotional impact and uncovered vulnerabilities associated with breaking bad news’.

Meeting patients’ emotional and information needs at diagnosis: a balancing act between disease, patient and organization-related factors

This theme explores participants’ experiences of balancing disease, patient and organization-related factors, along with the inherent challenges in breaking bad news, in order to provide an effective, empathic and patient-centred consultation.

All participants considered breaking bad news as a challenging yet crucial aspect of their role which they took seriously. It was generally acknowledged that receiving an MNDC diagnosis could be a life-changing process for patients, even the ‘*worst moment in their lives*’ (Participant 8, P8). Drawing from conversations with colleagues and a family member’s negative experience of being diagnosed with PD from a blunt neurologist, one participant supported the idea that suboptimal diagnostic experiences ‘*stick*’ with both patients and doctors (P6). Emphasising the importance of the diagnostic encounter, other participants mentioned that the quality of their interaction with patients at diagnosis could ‘*hugely*’ affect their future doctor-patient relationship (P3).

P8: ‘One thing I was told as a medical student is that patients forget your name, they might forget, you know, the stuff you say, the details, but they’ll remember how you were, how information was delivered and what you did afterwards.’

Participants agreed that because of the potential long-term ramifications of the experience of these consultations, it was crucial to deliver these diagnoses with empathy and sensitivity. However, this was considered the biggest challenge of breaking bad news by P1 who explained the paradox of having to give ‘*terrible*’ news in a gentle way.

P1: ‘It’s knowing how to do it right, that's the most challenging. How do you best give terrible news to somebody in a way that allows them to absorb the information without shutting down emotionally and without it being a traumatic experience. How do you give that information in a gentle way. Because that's in the end what you have to be, you have to be gentle, you're giving someone a massive blow. It's like trying to punch someone so hard to knock them out but you have to do it very gently.’

Adopting a gradual approach to giving the name of the diagnosis was described by all the participants as a way to mitigate this challenge. They usually started the consultation by asking patients to give their perspective, talk about their symptoms and share their thoughts on potential diagnoses. This helped doctors establish patients’ current knowledge and perspective and tailor the rest of the consultation. Using simple language and avoiding medical jargon, participants then explained the neurological basis of the patient’s symptoms while including some ‘*warning shots*’ (e.g., mentioning the motor nerve before disclosing an MND diagnosis). Participants believed that these warning shots ‘*softened the blow*’ (P2, P8) and prepared patients for the disclosure of the name of the diagnosis and minimised reactions of shock which could hamper information absorption.

However, despite participants’ gradual approach to breaking bad news, patients often reacted with shock or other intense emotions. It was generally agreed that it was important at that stage to give patients and families time and allow them to express these emotions of sorrow, despair, mourning or anger over the losses that an MNDC diagnosis might signal.

P8: ‘You can't judge or TELL people what the right reaction is because there isn't a right reaction, you just need to give them space to have the reaction and then be there. You shouldn't just rush out if at all possible. And even if everyone's sitting there not saying anything, you're being there, you’re available. And sharing that time is important.’

However, the same participant later in the interview also admitted that she was surprised when people had intense emotional reactions when receiving a PD diagnosis.

P8: ‘Sometimes, when somebody has quite a violent response to a diagnosis of Parkinson's disease that can be quite surprising for you and that’s awful, because we see so much Parkinson's that it’s almost one of the more benign diagnoses in terms of neurodegenerative diseases. So, that can sort of calibrate you when somebody is utterly devastated by a diagnosis of PD.’

As discussed earlier, diagnosis for participants was a rational and often expected outcome, so they were sometimes surprised when patients reacted with shock to bad news. This was more often the case for conditions such as PD or MS which participants even mentioned perceiving as a ‘*good news diagnosis in neurology terms*’ (P5) unless patients were young. PD and MS were considered less serious in neurology terms because of advances in the treatments available for these conditions and their better prognosis compared to HD and MND. In these instances, there could be a fundamental mismatch between neurologists’ and patients’ experiences, as professionals viewed the diagnosis from a biomedical lens while for patients this could nevertheless be a life-changing moment.

Overall, participants gave detailed responses in describing patients’ emotional reactions but provided less information on how they managed these reactions besides allowing patients’ time and space for emotional expression. For one participant (P4) emotional support was solely provided by nurses who were involved in the consultation and spent some time with patients and their families afterwards. Despite being prompted, other participants gave no information at all in terms of how they dealt with patients’ emotional reactions and needs or thought that these could not be managed.

P7: ‘Well, I don't know if you can really manage it, I think you just have to let them respond in the way that they're going to respond. If they're extremely devastated, you can't really manage it in a sense, you've just got to let them get on with it.’

This could potentially be an aspect of breaking bad news with which not all participants felt comfortable or confident, yet patients’ emotional reactions were considered useful in helping them structure the rest of the consultation and decide the nature and amount of information they should provide. Information-giving was generally perceived as a great responsibility by participants who wished to help patients understand their diagnosis but without further devastating them. Because of MNDCs’ progressive and often life-threatening nature, participants were ‘*wary of bombarding people with information*’ (P7). Even when patients explicitly asked questions on a sensitive subject such as their prognosis, participants often showed a reluctance to answer them. Some participants mentioned double-checking before imparting distressing information whereas another participant reported never answering patients’ with MND questions on life-expectancy. This appeared to be an exception to the overall patient-centred approach described in the interviews. Participants supported their practice either by explaining that the unpredictability around how MNDCs progress would not allow them to give an accurate prognosis or by expressing their intention to protect patients:

P8: ‘Patients can't know what they don't know. And you can't take away knowledge once it is given. You can't protect people from an outcome that may happen, but it's important to remember particularly near the beginning of the illness, that some knowledge can be so burdensome, damaging, that only providing it when it needs to be delivered or when the patient comes to you and says, ‘I've heard about this thing’. So I think knowing, gauging what people want to know, is very hard. Because what they say they want to know, might not be what they really want to know.’

Nevertheless, information at diagnosis was not always negative. As another strategy to soften the blow, participants offered patients reassurance by explaining what support was available and how their symptoms could be managed and signposting to other professionals, information sources and charities. P1 noted that, ideally, he wanted people to leave the consultation not just having understood their diagnosis, but also feeling ‘*a bit positive*’ and able to cope with it and gave information on current research on cures, current trials and alternative therapies. However, other participants believed that although they could promote optimism when delivering a PD or MS diagnosis, little scope existed for hope for HD and MND.

P4: ‘No, I don't give any hope in MND. I think it's unfair, because then they’d have an unrealistic expectation. I don't take away hope, but I don't give false hope. I try to encourage them to take each day at a time and do the things they want but I can’t give them hope.’

Time was an essential factor which affected the quality of the consultations that participants could offer. Neurologists believed that an ideal consultation should not feel rushed and should meet patient’s needs for both information and support. This was not a problem for P1 who worked in a specialist clinic that gave him the flexibility to spend as much time as needed with patients at diagnosis. However, for other participants, optimal diagnosis delivery was often hampered by organizational factors such as limited-service capacity and short time slots. Some participants reported having to break bad news in short, even 15-min, consultations.

P2: ‘These are fixed times; we don't have any options there. You're always clock-watching as a doctor the whole time. It's the biggest single negative of the job probably, the lack of time to do anything properly.

In general, based on their perceived hierarchy of the severity of different MNDCs, participants spent more time delivering MND and HD diagnoses compared to PD and MS. For example, P3 recounted investing one hour for the diagnosis of MND and HD and half that for PD and MS and P5 believed that even five to ten minutes was sometimes enough to convey a PD diagnosis. This seemed to be another exception to participants’ overall patient-centred approach. Also, knowing that a follow-up with the patient could be after a year, doctors who reached a diagnosis during a consultation sometimes had to break bad news to unaccompanied patients, a factor which also contributed to suboptimal diagnostic experiences. Other participants who also reported unrealistic time slots explained that they sometimes had to be ‘*resourceful*’ (P5), ‘*clock-watch*’ (P2), be ready for their clinics to overrun (P6) and even be ‘*naughty*’ by breaking rules and booking double appointments (P8) to provide an effective consultation.

Empathy makes the job harder: the emotional impact and uncovered vulnerabilities associated with breaking bad news

As discussed in the previous theme, participants emphasised providing a supportive consultation and maintaining an empathic approach in order to break bad news sensitively. Being empathic, however, was also a challenge that ‘*made the job harder*’ (P6) as participants had not only to deal with patients’ emotions but also their own. This theme explores how breaking bad news was emotionally experienced by participants, how it felt to be the bearer of bad news and the impact this task had on them.

Overall, participants acknowledged that breaking bad news for MNDCs was an emotionally burdensome task. Preparing to communicate an MND diagnosis or an unexpected MNDC diagnosis to young patients was an experience that sparked dread, causing fear and anxiety to even senior specialists. P8 reported that her ‘*state of mind and demeanour are very different*’ when she knew she had to break bad news. She experienced tension which started to build before the actual consultation and peaked right before she gave the name of an MNDC diagnosis:

P8: ‘There's always a moment, just before you say the name of the disease, where you feel terribly responsible. Like you've done it to them, that you've given it to them, I don't know if it’s just me. I don't know why it feels like in diagnosing that you own the disease, or sort of passing it through to them. That’s very sad’.

This seemed to be a recurrent theme among participants who, while not always expressing the feelings as overtly as P8, did use phrases indicative of a belief they were causing harm (P1: ‘*punching someone so hard to knock them out*’, P2: ‘*cutting someone’s life off*’, P3: ‘*dropping a bomb into the room*’, P6: ‘*wrecking someone’s life*’); clearly linguistically these emphasise the destructive nature of these diagnoses. These metaphors indicate that, for participants, communicating an MNDC diagnosis could feel like physically harming patients, a contradiction to their professional caring role and the ‘do no harm’ principle. Experiencing this contradiction could contribute to their reported feelings of responsibility and guilt when breaking bad news. Moreover, after diagnosis disclosure, participants were often emotionally challenged by having to witness patients’ reactions to the news, which could be understood as an immediate consequence of their actions. One participant vividly described this:

P6: ‘You can just see the bottom drop out of somebody's life in front of you and that's not nice.’

Being exposed to patients’ intense emotional reactions was experienced as an additional source of distress for professionals, eliciting sadness and sympathy for patients but also a feeling of powerlessness.

Interviewer: *‘So, in your opinion, what are the most challenging aspects of delivering a diagnosis for these incurable conditions?’.*

P3: ‘I think it's feeling powerless. People say that knowledge is power, but with incurable conditions, it doesn’t feel like that. So, I'm dragging them into giving them bad news and I can't really make anything better.’

This was particularly intense when delivering MND and HD diagnoses where a ‘*substantial treatment story was absent*’ (P5), in contrast with MS and PD which could usually be more effectively managed by disease modifying therapies and medication respectively.

Other participants, such as P1, had a milder emotional experience during a consultation of bad news but explained that he experienced the impact of breaking bad news after a consultation had finished.

P1: ‘When I'm in the interaction that's fine. It's afterwards, I find it incredible draining. During the consultation, I'm in the moment and I feel prepared. I understand people are going to react in different ways. […] I always wonder if I did it well, or if not, and how could I have done it better. I always feel completely exhausted, and I've noticed my communication skills plummet after this. I replay the consultation in my head and think 'could I have done it better?’, ‘was that the right thing?’.’

P1 could also observe feeling affected on cognitive and physical levels after diagnostic consultations, effects which were also common for other participants who mentioned feeling drained and experiencing migraines and stomach cramps.

Identifying with patients when breaking bad news was also perceived as an added difficulty which emotionally affected some participants. Delivering a diagnosis to patients with a similar age, gender or family circumstances to them was more upsetting for some neurologists. Breaking bad news in these cases ‘*brought it home*’ (P3) and acted as a reminder of participants’ own vulnerability and their own fears regarding the unpredictability of life. One participant discussed how becoming a parent made breaking bad news to parents more challenging:

P6: ‘It's impossible not to become a bit emotional sometimes, I don't know whether other clinicians would have said, but I found medicine a lot easier until I became a parent. So if I'm diagnosing Parkinson’s, and somebody’s age is X (participant’s age), and they've got a young family, that's harder for me now than it was. When I didn't have any kids and I was diagnosing Parkinson's in the X-year-old with kids that had less kind of direct kind of parallels with my life. I can't help but go there too. You know, well, what if that was me? Naturally your brain goes there. […] I think that's hard because you’ve got a clinic, you've got another four patients waiting. It's harder to get your brain back on track again, after a consultation like that.’

Identifying with patients was mostly conceived as a challenge. Due to their job demands, participants had to disengage from these emotions in order to move on to the next patient. While reflecting on the challenges of maintaining an empathic approach when breaking bad news, another participant admitted that it was easier for him to detach himself emotionally:

P2: ‘Well, you just look at them and imagine yourself in that seat. Just before I start talking, I try to flip the seats round and imagine me sitting there, but you can't get it too far because you have to carry on and be objective so there's a balance. It's very easy to just close yourself off and break some bad news and then walk out. It's not hard to do that. It's harder to open yourself up a bit.’

Some participants noted that being emotionally affected when breaking bad news could be an under-recognised aspect of their practice potentially because professionals were expected to put patients at the centre of every consultation. One participant stressed the need for his colleagues to recognise that they are ‘*only human*’:

P6: ‘I think, you just have to recognise you're only human, you're going to get affected and if you are affected, well, welcome, welcome to the human race!’.

Being human for these participants was associated with being vulnerable to emotions when breaking bad news. Even participants who believed it was normal to be emotional felt the need to highlight the fact that they had never been affected to the point of losing their objectivity and others reported that they experienced but did not express their emotions:

P3: ‘I have to stay there (after diagnosis disclosure) and probably manage my emotions by trying to feel a bit numb. So, using a kind of—"I'm watching myself giving bad news" rather than "I'm feeling myself giving bad news". I think that's probably a common technique that doctors use, so that you don't sort of cry badly, you can look empathetic and you can be open and you can be appropriately warm, but the only way to not feel that upset whenever people are really upset is to do this sort of 'imagine watching yourself', it's quite body-motion control technique.’

It is noteworthy that although—to some extent—all participants addressed the emotional aspects of being the bearer of bad news, some were reluctant to discuss this topic. This reluctancy could be as subtle as switching from first person to third person when addressing the emotional experience of breaking bad news or more prominent. One participant viewed breaking bad news just as part of his job (P5: ‘*This is what I'm paid to do*’) and it would be ‘*frivolous*’ for him to be anxious about it. However, this conceptualisation of his experience of breaking bad news changed as the conversation developed:

P5: Whenever you break bad news, it sort of reminds you of all the others. And it reminds you of your own predicament in life and of life, your fragility. You know, is it existential angst in a sort of way?’.

This is in alignment with accounts from other participants which suggest that breaking bad news was an experience which could uncover doctors’ professional and personal vulnerabilities. Reflecting and admitting these vulnerabilities and reliving the sad memories of previously breaking bad news could explain why some participants showed this initial reluctance in addressing emotional topics.

## Discussion

This was the first qualitative study to explore neurologists’ lived experiences of delivering an MNDC diagnosis, emphasising the experiential and emotional aspects of breaking bad news for these conditions. The analysis generated two main themes; the first theme focussed on participants’ patient-centred practice as a balancing act and the second theme focussed on the emotional experience and the emotional impact of breaking bad news.

Breaking bad news was perceived by participants as a challenging yet crucial aspect of their role. Patients’ varying information preferences and intense emotional reactions, time constraints, MNDCs’ incurable nature and a perceived limited scope for hope for conditions such as HD and MND were some of the challenges discussed by participants. Despite these difficulties and similarly to findings from other quantitative studies on neurologists’ perspectives on breaking bad news [20], neurologists reported good standards of practice, following a patient-centred approach and being sensitive to patients’ needs for information and support at such a critical time in their lives. However, patient studies have shown that a significant proportion of patients with MNDCs are still dissatisfied with how they received their diagnosis [20]. This study’s qualitative nature, participants’ in-depth reflections regarding their practice and the interpretative, inter-subjective understanding of participants’ accounts can help shed light on the seemingly contrasting findings between doctor and patient studies.

### Contrast between patient and professional perspectives

Inadequate information provision at diagnosis has been highlighted by patients with MNDCs as an aspect of diagnostic consultations that needs improvement [20]. Although participants in our study reported providing additional information and answering patients’ questions, this was not always possible due to short consultation times, especially for MS and PD diagnoses for which participants invested significantly less time. This could be experienced as abandonment by patients [39, 40]. Although not all patients with MNDCs want to receive prognostic information at diagnosis [41], some might experience dissatisfaction with the consultation when their autonomy is being compromised, their prognosis-related questions are left unanswered, or they feel they have to push for information [42].

Another frequently cited reason that contributes to sub-optimal diagnostic experiences for patients with MNDCs is professionals’ manner, specifically a blunt approach or a lack of empathy [20]. Although all participants in our study acknowledged the potentially life-changing nature of these diagnoses and the need to be sensitive, previous findings suggest a significant divergence in terms of how professionals and patients perceive and experience diagnosis delivery. For our participants, gradually explaining and naming a diagnosis was a rational process based on medical knowledge and clinical experience, especially when a diagnosis was suspected. Participants were therefore surprised when patients were still shocked and had ‘violent reactions’ to it. This was particularly the case for MS and PD, which were even considered ‘good news’ diagnoses by some participants since they were not directly life-threatening. Despite their emphasis on being empathic, MS and PD diagnoses were mostly viewed through a biomedical lens, not fully acknowledging the impact of these conditions on daily living and the stigma and identity disruption associated with them [43, 44]. The importance of appropriate responding by neurologists has been emphasised by patients [45] and is an area for which neurologists would like to receive further training [21, 29]. In this study a mixture of approaches were evident: some participants emphasised the need to be there for patients and share these emotional moments with them while for others it seemed that emotional support was mainly the nurses’ responsibility who could meet with the patient after the consultation.

### Understanding the emotional impact of breaking the news theoretically

Participants generally experienced breaking bad news as an emotionally burdensome and stressful task [21, 29], with the impact of stress extended beyond the actual consultation [14]. Apart from the distress derived from the task itself, participants arguably experienced moral distress too, a negative feeling evoked when clinicians cannot carry out what they consider to be ethically appropriate [46]. Moral distress can be induced by organizational restrictions common in healthcare institutions [47] such as those discussed among participants in this study: work overload, inadequate consultation slots and unavailability of quick follow-up appointments. These organizational restrictions arguably had an impact on both the standards of care professionals could offer at diagnosis but also their experience of breaking bad news (e.g., the stress of clock-watching). Acknowledging the experience of moral distress among doctors is critical as it has been identified as a risk factor for depression and job quitting and is associated with low job satisfaction [48].

Although empathy was recognised by participants as a prerequisite for effective breaking bad news consultations, empathy was also believed to ‘make the job harder’ by uncovering the vulnerabilities discussed above and making doctors susceptible to distressing emotions. Empathy has been recognised as a vital component of therapeutic relationships [49] and associated with a variety of positive outcomes for both healthcare professionals and patients [50–52]. Not surprisingly, breaking bad news protocols have emphasised its importance in meeting patients’ emotional needs [9, 30]. However, it is a common belief among doctors that empathy increases their vulnerability to patients’ suffering and might act as a risk factor for their well-being [53], yet only one out of ten studies in a systematic review supported this claim [54]. It has been proposed that the false belief that links empathy with burnout can be explained by a theoretical confusion between empathy and sympathy [55]. Empathy encompasses both emotional and cognitive domains which allow an individual to understand and feel others’ perspectives and experiences without losing the boundaries of the self [56, 57], whereas sympathy is the emotional identification with others which can lead to secondary traumatic stress and emotional over-involvement [58, 59]. Indeed, participants reported feeling sad and drained and showed sympathy when they were exposed to patient’s intense emotional reactions or had to deal with a particularly emotional case, which indicates that it was probably sympathy and not empathy which had the more intense emotional impact on them. This distinction is important as a fine difference exists between detachment and disengagement from patients, with increased detachment potentially perceived as apathy and lack of understanding by patients [60].

### Implications for practice and organizational change

In order to respond appropriately to patients’ information and emotional needs at diagnosis, neurologists firstly need to exercise their empathic approach by understanding patients’ reactions without imposing their own judgment regarding the severity of a diagnosis. Professionals should be guided by patients’ emotions, appropriately respond to these and offer tailored support and information, avoiding one-size-fits-all approaches (e.g., never promoting a sense of hope for MND or never discussing life expectancy at diagnosis). Neurologists can follow breaking bad news protocols such as the COMFORT model, which adopts a relational approach to breaking bad news that addresses the needs, expectations and desires doctors and patients bring in a consultation and can foster convergence between their perspectives [30]. Our findings also suggest that emotional vulnerability when breaking bad news should be recognised and not suppressed. Vulnerability theory suggests that vulnerability can be generative, promoting innovation, growth and fulfilment [61], but it requires self-awareness and self-care in order to be utilised in therapeutic relationships [62]. Appropriate training should, therefore, not just focus on breaking bad news but equip professionals with the appropriate skills of recognising their own vulnerabilities, managing their own emotions and reflecting on how these affect their practice [63]. Training should educate professionals on the fine differences between sympathy and empathy and detachment and disengagement, potentially utilising the concept of detached concern, a strategy that incorporates empathic concern and detachment in a dynamic way that both addresses patients’ needs without negatively impacting professionals’ well-being [64]. Apart from offering such training, it is fundamental that organizations make space for empathy and attend to doctors’ moral distress [55] by tackling severe staffing issues in neurology in the UK [65] and reconsidering current restrictions on time slots, especially for breaking bad news consultations.

### Limitations

Several limitations should be considered in terms of the present study’s methodology and focus. Firstly, we could argue that the neurologists who did take part might be more interested in the topic or more confident in their practice of breaking bad news so we understand that the results presented might not reflect all professionals. Secondly, despite attempts to ensure homogeneity, participants in our study practised in different types of settings (specialist/general) and different parts of the UK. This difference could potentially have a substantial effect on the experience of breaking bad news and we observed that participants who were able to offer longer consultations were also able to provide richer accounts compared to participants who practised in busy general hospitals. Thirdly, because of this study’s exploratory nature we chose to group four MNDCs together, however results could be more refined if these diagnoses were examined in separate studies, for example, to address the impact of different types of MND or MS on the experience of breaking bad news.

## Conclusions

Breaking bad news for MNDCs was a challenging task for neurologists who had to manage patients’ varied information and emotional needs, while also managing their own emotions, a heavy workload and time restrictions. The IPA approach allowed an exploration of the intricacies of the experience of breaking bad news and helped highlight how participants’ practice was shaped by their perspectives and how the task uncovered their personal and professional vulnerabilities. Exploring the lived experience of being the bearer of bad news in the context of MNDCs also helped explain the observed differences between studies of doctors’ and patients’ perspectives on diagnosis delivery and suggest ways to support professionals with this task and eventually optimise the patient experience.

## Supplementary Information

**Additional file 1.** Supplementary Material 1.

**Additional file 2.** Supplementary Material 2.

## Data Availability

The raw data that support the findings of this study are not publicly available because they include information that could lead to the identification of participants. Anonymised transcripts are available from the corresponding author on reasonable request.

## Abbreviations

MNDC: Motor neurodegenerative condition
IPA: Interpretative phenomenological analysis
