# Supplementary material for: Neurologists’ lived experiences of communicating the diagnosis of a motor neurodegenerative condition: an interpretative phenomenological analysis
Source: BMC Neurol. 2023 May 3;23:178. doi: 10.1186/s12883-023-03233-3 (PMC10155430; doi:10.1186/s12883-023-03233-3)
Supplement: Supplementary file 1 — Additional file 1. Supplementary Material 1. [file 12883_2023_3233_MOESM1_ESM.docx]

**SM1: Interview Schedule**

| Question | Rationale | Prompts |
| --- | --- | --- |
| Can you tell me how you have come to work in this context and this line of work? | Warm-up, demographic questions which will also provide context to the rest of the interview | How long are you in practice? How old are you? Where were you trained?  What kind of diagnoses do you give? |
| What are your experiences with communicating with patients in this context in general? | A warm-up, more general question which will also provide context and will reveal physicians’ general attitudes towards doctor-patient communication | How important do you think doctor-patient communication is? |
| So, let’s say that you have to deliver a diagnosis for a MNDD. How do you prepare yourself before the consultation? | Inspired by the SPIKES protocol and the Goals-Plans Action (GPA) theory, this question aims to explore physicians’ practice regarding the preparation of the consultation and the goals they set. | What kind of goals do you set for the consultation?  What do you think the main goal of diagnosis delivery is?  How do you feel and what are your emotions at this point? |
| What is the usual practice in your organisation regarding the setting where diagnosis delivery takes place? | Checking the setting where diagnosis is delivered and the provision of privacy, a basic standard of good practice | Is privacy always guaranteed?  Did you ever have to deliver the diagnosis through the phone? |
| Are there any other people usually involved in the consultation? | Checking the involvement of other people in the consultation as it has been shown that this can be helpful for the patient, although challenging for the professional | …such as patients’ family or a nurse  Do you think it is helpful for you or the patient to include others in the consultation? |
| How much time do you usually dedicate to a diagnosis delivery consultation for a MNDD? | Short consultations are a problem according to patient studies | Do you dedicate a different amount of time for different MNDDs?  Do you think this is enough for patients to get all the information needed at that point? |
| How do you start the consultation? What would you usually say? Can you narrate for me how a typical consultation would go? | Invitation step in the SPIKES protocol  Exploring their Actions according to the GPA theory | Do patients usually know they are about to receive bad news?  Do you try to give some warning signs that you are going to break bad news? |
| What is your emotional state like when you are giving the name of the condition and you see the patient’s reaction? | Lived experience, exploring their emotions |  |
| What are patients’ usual reactions to the news? | Explore their empathy through assessing they ability to identify patients’ emotional reactions and respond to them  An opportunity for them to provide a narrative | How do you respond to their reactions?  Do you give them time to express their emotions?  Do you remember any occasions when the patient had a very strong reaction when they received the diagnosis?  How do you react if a patient starts crying?  Has anyone ever got an angry reaction to the news? How did you manage this?  What are usually the reactions of the family? How do you manage them? |
| Have any experiences of breaking bad news particularly memorable for you? | Lived experience, giving specific examples and providing a narrative | Either positive or upsetting  Can you talk to me about it? |
| How do you check patients’ information preferences? | Information provision is a significant aspect of breaking bad news according to literature and it provides the foundations for shared-decision making | Do you withhold any kind of information when you are delivering a diagnosis for a MNDD?  How do you decide what kind of information to provide?  When talking about treatment, do you enable the patient to express their personal needs and preferences? |
| Let’s say that you have to deliver the diagnosis of MS to a woman in her 30s with 2 children, how does this make you feel? | An attempt to approach the lived experience of breaking bad news via this hypothetical scenario | How do you go about doing this? How would you describe your experience in taking on such a task?  Can you describe any somatic feelings?  Do patient factors such as their age affect your emotions and how you deliver the diagnosis? |
| Have you ever been emotionally affected after a breaking bad news consultation? | Exploring the emotional impact of breaking bad news | How did you feel? |
| How is a breaking bad news consultation different than other consultations? | Exploring their perspectives on breaking bad news | Do you experience it in a different way? Do you think it is a difficult task? Why? Do you think it is a stressful task? Why? |
| How does diagnosis delivery differ among MNDDs? | Exploring their perspectives specifically for MNDDs and differences among them | Any illness-related factors that you take into account when delivering the diagnosis? |
| What are the most challenging aspects of delivering a diagnosis for an MNDD? | Exploring their perceptions on what are the difficulties in breaking bad news for MNDDs | In case they do not provide enough information to the open-ended question, examples will be given by the interviewer on which the participant can elaborate on (e.g. incurable nature of the conditions, responding to patients’ emotions) |
| Can you give me an example of a consultation that went particularly well? | Exploring an example of a positive experience and checking what made it positive | How do you feel after diagnosis delivery has been particularly effective? |
| Do organisational/healthcare system factors affect the way you deliver a diagnosis for an MNDD? | Examine contextual factors which affect neurologists’ practice | In case they do not provide enough information to the open-ended question, examples will be given by the interviewer on which the participant can elaborate on (e.g. time constraints) |
| In general, how would you self-assess how well you communicate the news for a motor neurodegenerative condition? | Checking how neurologists self-assess their ability to deliver such a diagnosis effectively | How confident are you that patients leave the consultation having taken in all the information relevant to them at that point?  In general, how satisfied you think your patients are with the way the diagnosis is delivered? |
| What kind of education and training have you received on communication with patients? | Examining their education and training needs | Have you received training specifically for breaking bad news?  Do you follow any specific guidelines for breaking bad news? If no, do you think it would be valuable for your organisation to follow a specific set of guidelines?  Would be interested to receive further training on breaking bad news?  How has your practice on breaking bad news changed over the years? |
