# Supplementary material for: Neurologists’ lived experiences of communicating the diagnosis of a motor neurodegenerative condition: an interpretative phenomenological analysis
Source: BMC Neurol. 2023 May 3;23:178. doi: 10.1186/s12883-023-03233-3 (PMC10155430; doi:10.1186/s12883-023-03233-3)
Supplement: Supplementary file 2 — Additional file 2. Supplementary Material 2. [file 12883_2023_3233_MOESM2_ESM.docx]

| Reflection – Initial ideas  **SM2. Audit Trail**  **1. Coding example** | Transcript excerpts | Coding  Descriptive/interpretative/linguistic |
| --- | --- | --- |
| *Maybe there isn’t a perfect way to deliver such diagnoses that’s where support, empathic and compassionate practice could be fundamental?*  *The participant has been reluctant to discuss the emotional aspect of delivering these diagnoses up to this point – what he says here might explain why (see coding) – I should have asked why he believed that.* | I: So, what are the most challenging aspects of delivering a diagnosis of MND?  P: Is knowing how to do it right, that's the most challenging. How do you, how do you best give terrible news to somebody in a way that allows them to absorb the information without shutting down emotionally and without it being such a traumatic experience that they can't, they just re-live it it or they can't even think about it? It's that, how do you give that information in a gentle way. Because that's in the end what you have to be you have to be gentle, you're giving someone a massive blow. It's like trying to punch someone so hard to knock them out but you have to do it very, very gently.  I: So ideally, how do you want the patient to leave the room?  P: I want them to leave, having absorbed the information in a state of mind where they can deal with it. I want them to leave feeling positive. So, a lot of what I do is, after giving the terrible diagnosis, tell them what we can do, and telling them that there's an army of researchers trying to support them, and, you know, I don't know how long it'll be before we find a cure, but everyday we're closer, obviously. And I try to give them hope and I help them with things like alternative therapies, you could go here. And there's these clinical trials, and we do this research...  I: So, you give them some treatment and management options…  P: Yes, yeah. The other thing, as I say, we have a multidisciplinary team, so they know they're supported. And I make it explicit that we don't want them to feel abandoned. And that the purpose of our clinic is that they can ring or contact us at any time for any support they need.  […]  Have you ever been emotionally affected after consultations when you had broken bad news?  Yes.  How did you feel?  Well, sad. Yeah. I mean, I don't know what more I can say. You know, I could give you a sort of florid series of metaphors about my emotional status, but I don't think it would help very much.  Oh, why not? You can just give me one.  Okay. Well, maybe I can't, but let me just think. I suppose it's a bit like going to funerals. I don't know if you've been to many. But you know, whenever you go to a funeral, it reminds you of all the others that you've ever been to, including those of your nearest relatives. And, whenever you break bad news, it sort of reminds you of all the others. And it reminds you of your own predicament in life and of life, fragility.  See? That's not a metaphor. That's really interesting!  It's not, it's not! That's true. That's true.  So it's kind… there's this sense of sorrow, kind of.  Sorrow. Yes, that's true. You know, is it existential angst in a sort of way? Yeah. | Knowing how to give ‘terrible’ news without hampering patient’s information absorption, shutting them down emotionally and contributing to a traumatic experience is the most challenging aspect of BBN – places the challenge of BBN on ‘knowing’ rather than the ‘doing’, implies BBN is a process that can be approached in different ways and primarily requires knowledge and potentially experience - he’s sensitive to the immediate impact the diagnosis can have on patients.  Uses metaphors to describe the paradox of having to give such bad (‘terrible’) news in a gentle way: ‘being gentle when giving someone a massive blow’ & ‘punching someone so hard to knock them out but doing it gently’. The use of these verbs and metaphors illustrates the potentially destructive nature of an MND diagnosis and also imply a sense of responsibility for the diagnosis, personal agency – it sounds like BBN for MND is a lose-lose situation, where neurologists will inevitably distress patients through diagnosis communication – however, the need to be gentle and sensitive is emphasised  Wants people to leave the consultation having absorbed information, being able to cope with it and feel positive. After giving the diagnosis he tries to instil hope by informing patients about current research on cures, current trials and alternative therapies they can try  Balancing bad news by also promoting some sense of hope for potential research advances regarding therapy. Being well-informed but still feeling a bit positive as ideal outcomes of an effective BBN consultation.  Informing patients about the MDT support available to them and the availability of the clinic for whatever support patients might need – Another positive message he includes during BBN consultation: providing reassurance about the availability of long-term support, acknowledges the potential feelings of isolation and helplessness that receiving an MND diagnosis might trigger  Feeling sad after breaking bad news – says he doesn’t know what more to say other than feeling sad. – reluctance to elaborate on the emotional impact of BBN  He can elaborate on his emotional state after BBN using metaphors but does not believe this would be helpful – potentially views his own emotional experience of BBN as being insignificant or he wants to avoid elaborating on experiences that might have been distressing for him. Throughout the interview he has focussed on the information that needs to be imparted and has viewed emotional aspects of these consultations as secondary to the information exchange.  Breaking bad news is a sad process that feels like going to funerals, it reminds him of all the times he’s broken bad news and reminds him of his own predicament in life, his fragility – The funeral metaphor might imply a general feeling of sorrow surrounding the process of giving bad news. Breaking bad news triggers memories from all similar past consultations and also acts as a reminder of his own fragility - these add to the sadness experienced when conducting this task but also make it personal for the doctor as well, exposing his own vulnerability and mortality.  The sorrow experienced when delivering these diagnoses could trigger existential angst – beyond the experience of distressing emotions, being the bearer of such bad news can trigger philosophical questions about life |

**2. Example of an interpretative summary from an individual interview**

**Interpretation both at coding level and when writing these narrative summaries**

**Example 1.** *Hierarchy of neurological conditions*

| Narrative summary | Codes | Examples of supporting quotes |
| --- | --- | --- |
| When she was asked about her general experience in communicating the MNDD diagnosis, the participant presented a hierarchical ordering of neurological conditions, based on how manageable and directly life-threatening they are. The perceived severity of an MNDD seemed to influence her perceived difficulty of diagnosis delivery, her emotional experience of breaking bad news and some parameters of her practice (such as the amount of time invested for the consultation). At the bottom of her hierarchy of neurological conditions was MS. Due to advances in available disease-modifying treatments the participant could offer a better prognosis to patients which made it one of the ‘easier diagnosis to give’. She reported telling patients that an MS diagnosis is ‘*actually good news*’, implying that it could be something worse than MS. Her professional knowledge and clinical perspective allow her to contrast and compare MS to other, more severe conditions, [yet from the patient’s perspective MS could still be a disruptive diagnosis which they do not necessarily compare with other more severe ones]. Similarly, she found PD to be ‘*nowhere near the worst thing*’ she has to diagnose, mostly because of its chronic nature (versus conditions with limited life expectancy) and the variety of options she could offer patients (medications, physiotherapy, rehabilitation etc.) to help manage their condition. However, she found diagnosing young onset Parkinson’s difficult because of how upsetting and life-changing the diagnosis can be for young people. Moving up the ‘hierarchy’, she believes that HD is one of the worst conditions to diagnose because of the ‘often-huge implications’ on a family level and the fears regarding the hereditary nature of the condition. On a more positive note, unlike the rest of MNDDs, an HD diagnosis does not usually come out of the blue, patients might have known for years, so breaking bad news is more challenging when the patient and their family did not know about a positive gene testing for HD. According to the participant, MND was the diagnosis she struggled with the most due to its *‘bleak*’ nature associated with a complete lack of positive aspects she could discuss with patients. When breaking the bad news of MND, she feels that she cannot offer much for patients, but at the same time she had to deliver a significant amount of distressing information. The hierarchy that she presented can also explain why the participant reported spending 1-1.5 hours to BBN for MND and HD, but half of that for PD and MS. | Because of advances in available treatments, BBN in MS has changed massively for the better since she feels she can discuss a better prognosis  Views MS as ‘good news’ which she might also mention to patients  She tells MS patients that the chance of being in a wheelchair 5 years post-diagnosis, has gone from 50 to 5%  Giving an MS diagnosis can be a positive discussion that includes treatment options  MS is now one of the easier diagnoses she gives - ‘Easier’ not ‘easy’ might imply that it’s still difficult nevertheless  People know about PD but dread it coming with a preconception that it is incurable, and nothing can be done about it, although she states this is not entirely true as PD for her is ‘nowhere near the worst thing she has to diagnose’. [mismatch between her and patients’ experiences]  Discussing ways to manage PD make it an ‘easier’ diagnosis.  She presents a ‘hierarchy’ of neurological conditions based on their severity which affects how difficult diagnosis giving is for her – based on her clinical knowledge and experience, potential divergence to the patient’s experiences?  More difficult to deliver a young onset diagnosis for PD as it can be life-changing and upsetting  The often-huge implications of a HD diagnosis on a family level makes it one of the worst conditions to diagnose  Struggles the most with giving an MND diagnosis because it is bleak – associated with the lack of positive aspects of it, a diagnosis is difficult when she feels she can’t offer much to the patient – ‘bleak’ emphasises the hopless and destructive nature of this diagnosis  MND diagnosis as ‘awful news’ - Besides giving an MND diagnosis, she has to make people think about making decisions about the future, even their end-of-life care – bad news beyond just naming the condition  Books double slots to deliver an MND diagnosis – more serious diagnosis, more info to be shared  For most BBN consultations she spends 1-1.5 hours and half of that for PD and MS | ‘*Communicating with patients with a diagnosis of MS has changed massively in the last 15 years. And that's probably because we're able to offer a much better prognosis.*’  ‘*I say show them the scans, I say, “There are multiple, these are called scleroses and this is MS, had you thought about that?”, and they say “yes” or “no” and then I'll say, “This is what this means, actually this is quite good news”, etc.*’  ‘*So, actually, I find the diagnosis of MS is much easier, it's positive, we can talk to them about treatment*.’  ‘*A Parkinson's disease diagnosis is still nowhere near the worst thing that I have to do, I can often say to people, “We can't cure this, but we often can manage it really well and there are lots of things that we can try to help you get better”.*’  ‘*The big different for Huntington's disease is usually then the family will come to the diagnostic interview and, erm, everybody's invested interest in it. And I once diagnosed somebody with Huntington's disease at the age of 82. And she had a lot of children and grandchildren so, the implications were huge, really. So, Huntington's disease tends to be one of the worst because of the wider view*.’  ‘*But, of all the things I diagnose, MND is the thing that I find hardest to discuss because it is so bleak and erm... I can't find many positives to offer at all.*’  *‘If we're dealing with MND, first of all, I'll give myself a double slot to break that bad news.*’ |

**3. Theme titles of all interpretative summaries develop from each interview**

**P1:**

- I have a way of doing it: a gradual approach to breaking bad news
- Punching someone but gently: the paradox of imparting distressing information in a sensitive way
- After giving the name – assessing patient’s info needs and dealing with patients’ reactions
- Being part of a specialist MND clinic makes everything easier

**P2:**

- Leave a piece of you in the room or be a robot
- Clock watching
- Softening the blow

**P3:**

- Hierarchy of neurological conditions
- ‘*I’m still human*’
- Assessing information needs and tailoring information giving
- Providing a supportive consultation

**P4:**

- It’s sad but you have to be strong
- Acknowledging patients’ need for support during and after the consultation – ‘Hope would be unfair but live for today’

**P5:**

- Focusing on aiding patient understanding
- ‘The most central thing I do’ – what makes it emotional and difficult

**P6:**

- The importance of providing a tailored consultation when breaking bad news
- ‘You’re only human’ / ‘Welcome to the human race’: the emotional toll and emotional triggers of being the bearer of bad news

**P7:**

- A patient-centred approach to information giving
- Reluctancy in talking about the emotional experience of breaking bad news

**P8:**

- Sharing the silence: allowing people to express their emotions
- ‘You’re telling a fellow human being they have a horrible disease’ – the emotional experience and impact of breaking bad news.
- Beyond giving a name: the challenges of knowing how far to go with information giving at diagnosis in the context of MNDDs
